# The growth patterns, hemoglobin pretransfusion, serum ferritin and bone age in thalassemia major patients

**DOI:** 10.1186/1687-9856-2013-S1-P41

**Published:** 2013-10-03

**Authors:** Made Arimbawa, Ketut Ariawati

**Affiliations:** 1 Departement of Pediatrics, Medical Faculty, Udayana University/Sanglah Hospital, Denpasar-Bali, Indonesia

## 

Despite frequent blood transfusions combined with chelation therapy lead to an improved rate of survival, endocrine disorders related to secondary hemosiderosis such as short stature, delayed puberty and hypogonadism are major problems in adolescent children with thalassemia major. The aim of this study was to know the description of height, growth velocity, bone age, hemoglobin pretranfusion level and serum ferritin in thalassemic patients.

The retrospective study of children hospitalized in Pediatrics ward Sanglah Hospital Denpasar from December 2010-February 2011.

Fifteen subjects were diagnosed as thalassemia major, aged between 1.91 years - 13.5 years; 7 boys and 8 girls. Two children aged less than 3 years and 7 children had entered puberty. All patients had to undergo iron chelation therapy deferioksamin with inadequate quality. Short stature was found in 4 children (26%), all with growth velocity of <5 cm/year. Clinically 1 person categorized as delayed puberty. Mean hemoglobin pretranfusion levels can be maintained ≥ 8 mg/dl (10), the remainder (5) has an average hemoglobin below 8 mg/dl. Four children with serum ferritin over 3000 ng/ml, all with short stature. In the radiological evaluation (bone age) 5 children have delayed bone age.

Our study suggests that thalassemic patients, short stature is found in 26% cases and all of them have entered the age of puberty. All patients with short stature has serum ferritin levels >3000 ng/ml.

